# Single-operator cholangioscopy in the diagnosis and treatment planning of hilar intraductal papillary neoplasia of the bile duct: a case report

**DOI:** 10.1016/j.igie.2026.03.003

**Published:** 2026-03-12

**Authors:** Dario Gambaccini, Filippo Ovidi, Delio Stefani Donati, Veronica Natali, Arianna Venturini, Gherardo Tapete, Valeria Bolognesi, Andrea Pancetti, Pierfrancesco Visaggi, Antonio Grosso, Emanuele Marciano

**Affiliations:** 1 Digestive Endoscopy Unit, Department of Abdominal Surgery and Urology, Azienda-Ospedaliero-Universitaria Pisana, Pisa, Italy; 2 Faculty of Medicine and Surgery, Gastroenterology Unit, Department of Medical and Surgical Specialties, University of Pisa, Pisa, Italy

## Abstract

A 53-year-old man presented with abdominal pain and cholestasis with normal bilirubin. Computed tomography and magnetic resonance imaging/magnetic resonance cholangiopancreatography suggested a perihilar stricture but could not exclude left-duct extension, and initial multidisciplinary review deemed the case unresectable. Endoscopic retrograde cholangiopancreatography with peroral single-operator cholangioscopy (SOC) obtained biopsy specimens, but the stricture was not traversable; repeat multidisciplinary review pursued antegrade mapping. A left percutaneous transhepatic drain was placed; 11 days after percutaneous drain placement, antegrade percutaneous SOC (pSOC) mapped right duct involvement and confirmed left hepatic duct sparing, revising resectability. The patient underwent associating liver partition and portal vein ligation for staged hepatectomy followed by extended right hepatectomy. Pathology showed intraductal papillary neoplasm of the bile duct with high-grade dysplasia and microscopically negative margins (R0); no adverse events occurred. Combined peroral and pSOC can enable curative surgery when imaging and endoscopic retrograde cholangiopancreatography are inconclusive.

## Introduction

Resectability of malignant-appearing perihilar strictures hinges on defining longitudinal ductal spread. Computed tomography (CT) and magnetic resonance imaging/magnetic resonance cholangiopancreatography (MRI/MRCP) may leave contralateral extension uncertain, leading to conservative judgments. Digital single-operator cholangioscopy (SOC) offers direct visualization and targeted biopsies, improving diagnostic accuracy and interobserver agreement in the evaluation of indeterminate biliary strictures,^1^, ^2^, ^3^ but can be limited by high-grade hilar stenoses. In this case, incomplete mapping during endoscopic retrograde cholangiopancreatography (ERCP) left contralateral extension uncertain, and the stricture was initially deemed unresectable. Uniquely, combined peroral and antegrade percutaneous SOC (pSOC) was used not only for diagnosis but also to define ductal extent and determine resectability criteria, overturning the initial assessment.

## Case description

A 53-year-old man with no comorbidities and no prior surgical history presented with 1 month of right upper abdominal pain and biochemical cholestasis (γ-glutamyl transferase 1114 U/L [normal range, <70 U/L]; alkaline phosphatase 1004 U/L (normal range, <80 U/L]; aspartate aminotransferase 72 U/L [normal range, <40 U/L]; alanine aminotransferase 54 U/L [normal range, <40 U/L]; carbohydrate antigen 19-9 43 U/mL [normal range, <9 U/mL]), with total bilirubin within the normal range (<1.2 mg/dL). He was admitted for evaluation. CT (Fig. 1) and MRI/MRCP (Fig. 2) revealed a malignant-appearing perihilar stricture with indeterminate extension toward the left hepatic duct; at the initial multidisciplinary review, the case was deemed unresectable because of presumed bilateral involvement.

**Figure 1:**
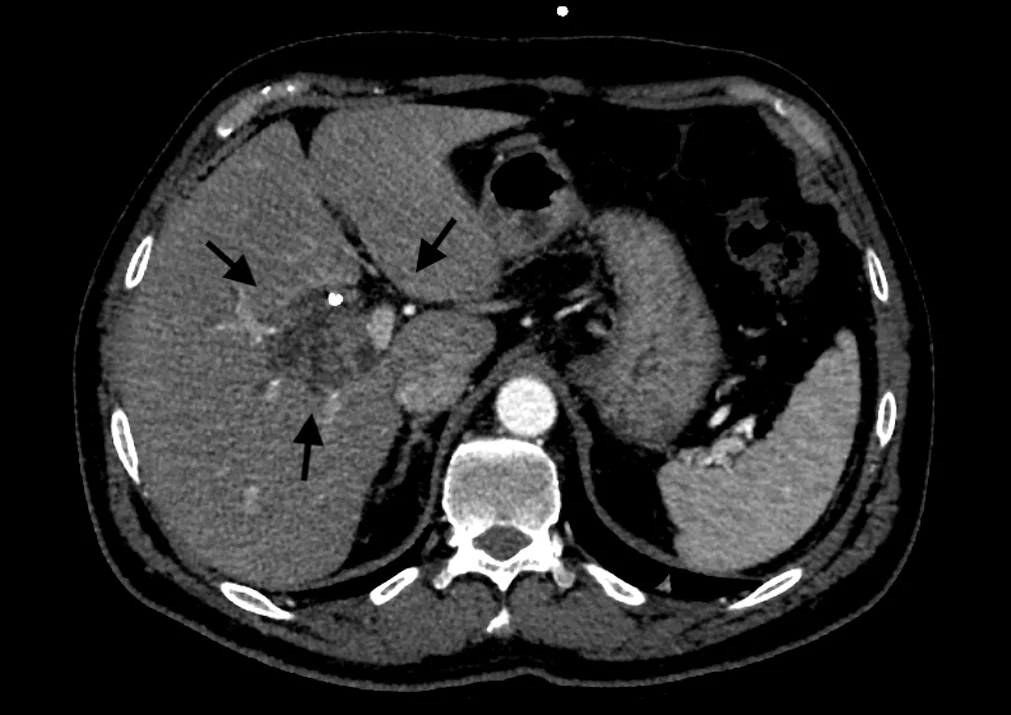
Preoperative contrast-enhanced computed tomography (*axial view*) showing a malignant-appearing perihilar biliary lesion/stricture (*arrows*) with associated upstream biliary dilatation (*predominantly right sided*).

**Figure 2:**
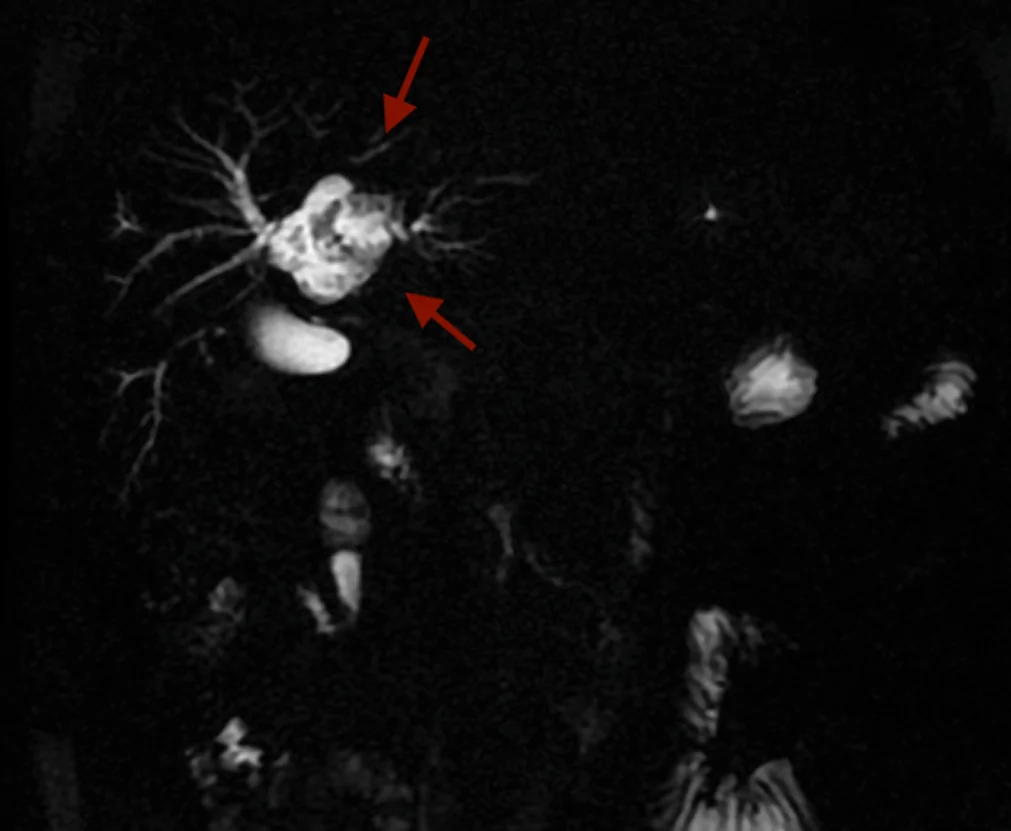
Preoperative magnetic resonance cholangiopancreatography demonstrating an intraductal perihilar lesion at the biliary confluence, causing hilar obstruction; *arrows* highlight the lesion and its indeterminate extension toward the left hepatic duct.

ERCP was performed using a therapeutic duodenoscope (Fujifilm ED-580XT, working channel 4.2 mm; Fujifilm Corporation, Tokyo, Japan) for diagnostic purposes (targeted biopsies and precise lesion mapping), without biliary stent placement. Biliary cannulation was achieved with a 0.035-inch hydrophilic guidewire (Dreamwire; Boston Scientific, Marlborough, Mass, USA) through a cannulating sphincterotome (Autotome RX; Boston Scientific). Cholangiography demonstrated a tight hilar stricture with upstream right-sided ductal dilatation; the biliary confluence and left hepatic duct could not be adequately opacified/assessed. Transpapillary digital SOC (SpyGlass DS II–SpyScope DS II catheter; Boston Scientific) at the hilum provided direct visualization of irregular, papillary/villous intraductal tissue and enabled cholangioscopy-guided forcep biopsies (SpyBite Max, 1.0-mm jaw; Boston Scientific). Histology from the cholangioscopy-guided biopsy specimens was compatible with gastric/intestinal-type intraductal papillary neoplasm of the bile duct (IPNB; cytokeratin 7 positive [CK7+], mucin 5AC positive [MUC5AC+], mucin 6 positive [MUC6+], mucin 1 negative [MUC1−], mucin 2 positive).^4^ The tight stricture could not be traversed into the right system (Figs. 3 and 4); accordingly, SOC could not be advanced beyond the stricture, and the biliary confluence and left hepatic duct could not be assessed or accessed during the initial ERCP. Thus, right-sided involvement was suspected but could not be definitively mapped, and contralateral (left-sided) involvement could not be excluded.

**Figure 3:**
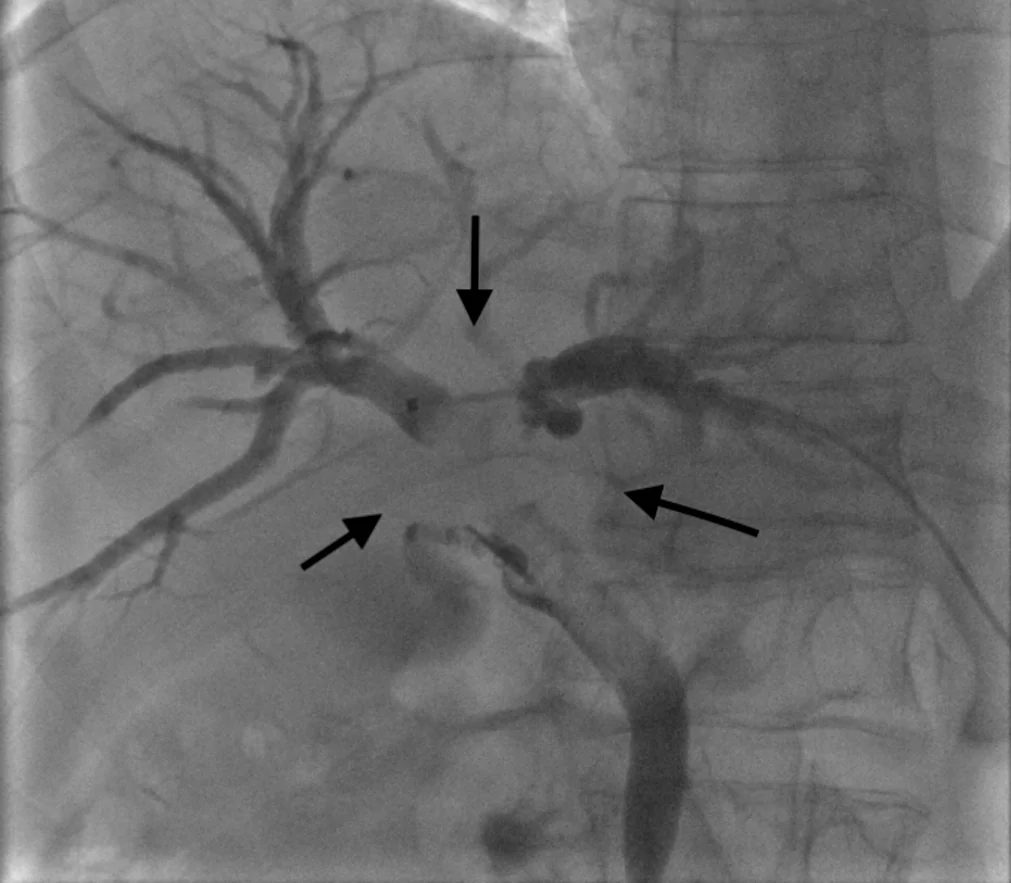
Endoscopic retrograde cholangiopancreatography cholangiogram showing a tight hilar stricture at the biliary confluence (*arrows*) with preferential opacification/dilatation of the right intrahepatic ducts; the biliary confluence and left hepatic duct are not adequately opacified/assessed.

**Figure 4:**
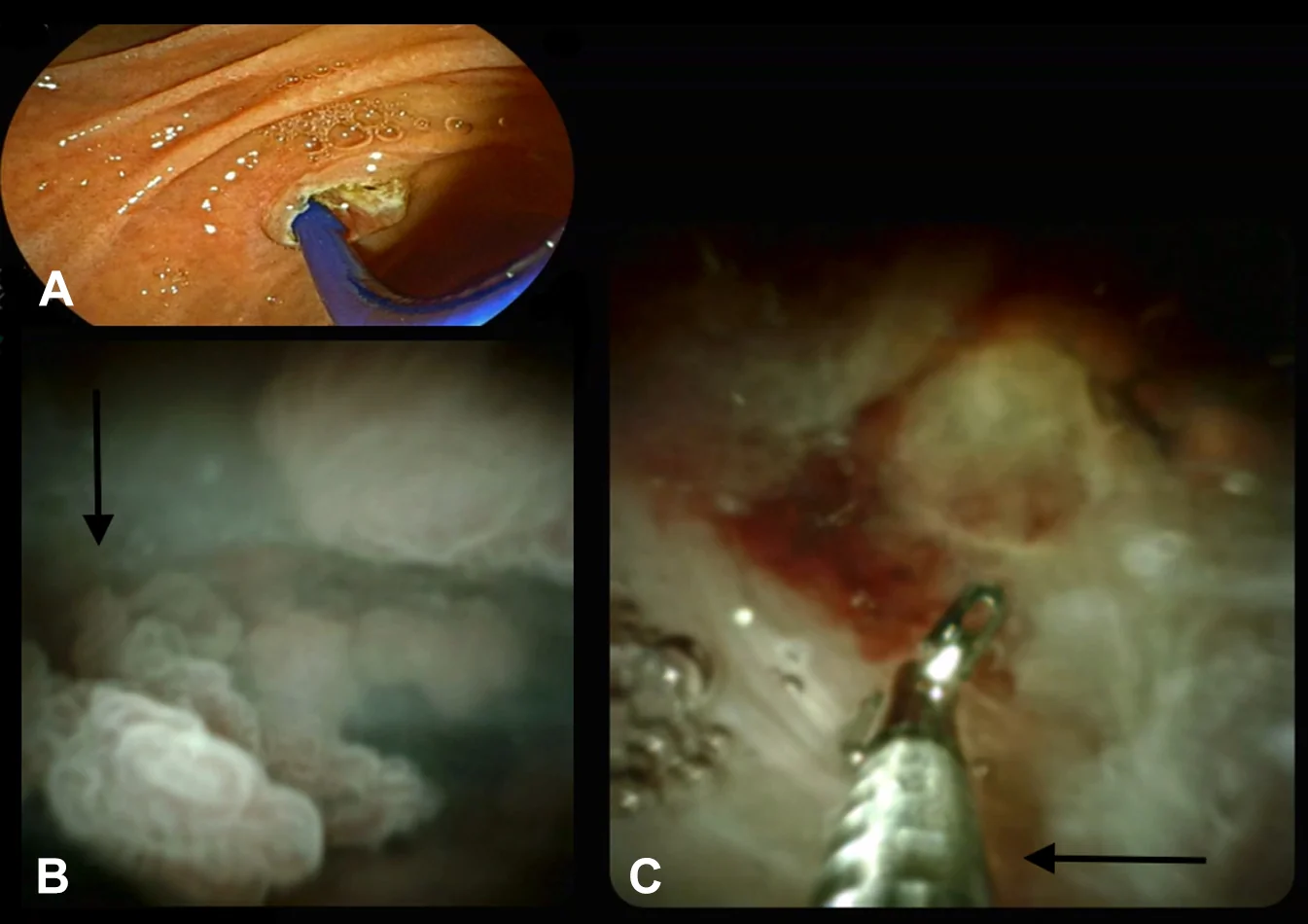
Tissue sampling during peroral digital single-operator cholangioscopy. **A,** Duodenoscopic view showing transpapillary introduction of the cholangioscope. **B,** Cholangioscopic view showing papillary/villous intraductal tissue at the hilum (*arrow*). **C,** Close-up cholangioscopic view showing the papillary/villous lesion and cholangioscopy-guided biopsy with dedicated forceps (*arrow*).

During the same admission, the day after ERCP, the case was discussed at the multidisciplinary team review. Because ERCP had not allowed complete ductal mapping, the multidisciplinary team requested more-precise remapping to better define resectability; therefore, interventional radiology (IR) placed a left percutaneous transhepatic drain to initiate fistulous tract maturation (serially dilated to 12F). Eleven days after percutaneous drain placement, antegrade pSOC (SpyGlass DS II; Boston Scientific) through the matured 12F transhepatic tract mapped involvement of the right intrahepatic ducts and confirmed no involvement of the left hepatic duct (Fig. 5) (Video 1, available online at www.igiejournal.org). These findings overturned the initial unresectable assessment and prompted a curative plan. No adverse events occurred.

**Figure 5:**
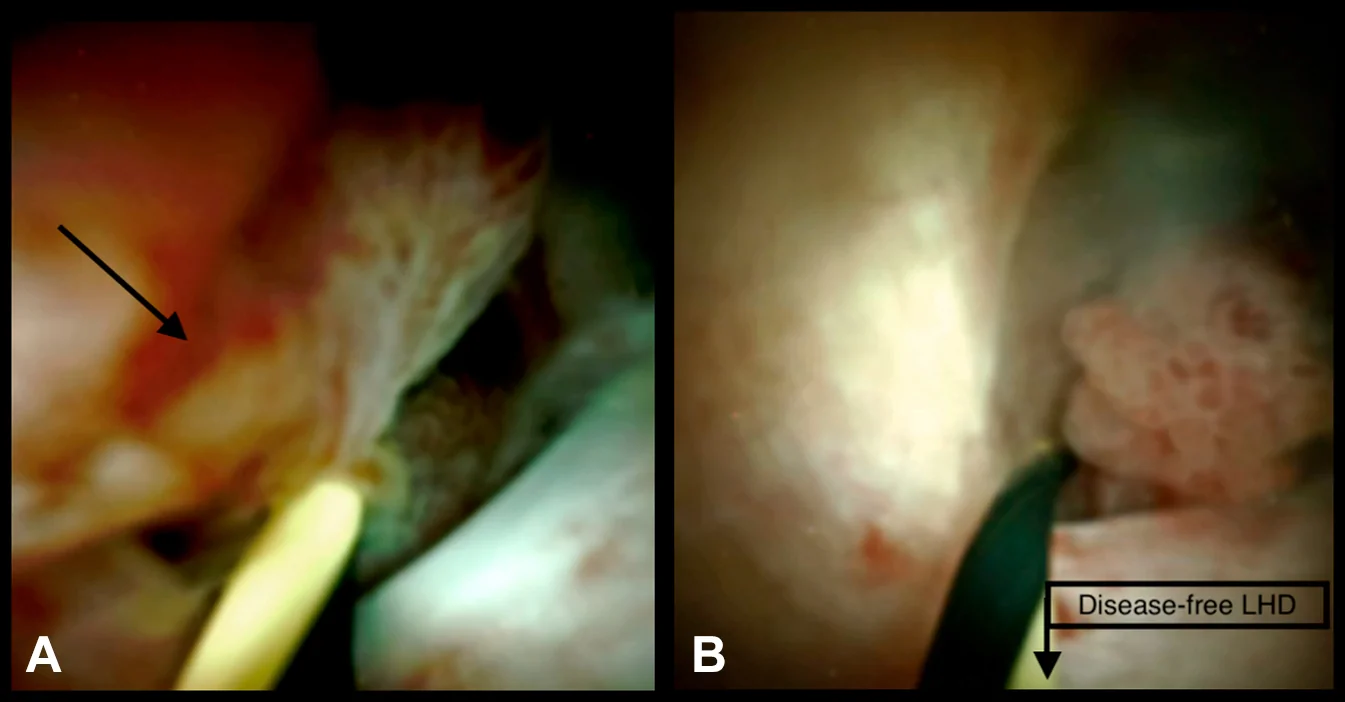
Antegrade percutaneous single-operator cholangioscopy performed through a matured left transhepatic tract. **A,** Cholangioscopic view of a papillary/villous intraductal lesion consistent with intraductal papillary neoplasm of the bile duct (*arrow*). **B,** Inspection of the left hepatic duct (LHD) showing no endoluminal lesion; the *label/arrow* indicates a disease-free LHD.

Given the anticipated small future liver remnant, the team performed a staged approach: stage 1, associating liver partition and portal vein ligation for staged hepatectomy (ALPPS) to induce hypertrophy, followed by stage 2 extended right hepatectomy. The postoperative course was uneventful. Final pathology showed IPNB with high-grade dysplasia and microscopically negative margins (R0) (CK7+; MUC5AC+; MUC6+; MUC1−). The patient remained well on surveillance with annual MRI (Fig. 6). The patient reported relief that the additional cholangioscopy made curative surgery possible and felt well supported throughout treatment.

**Figure 6:**
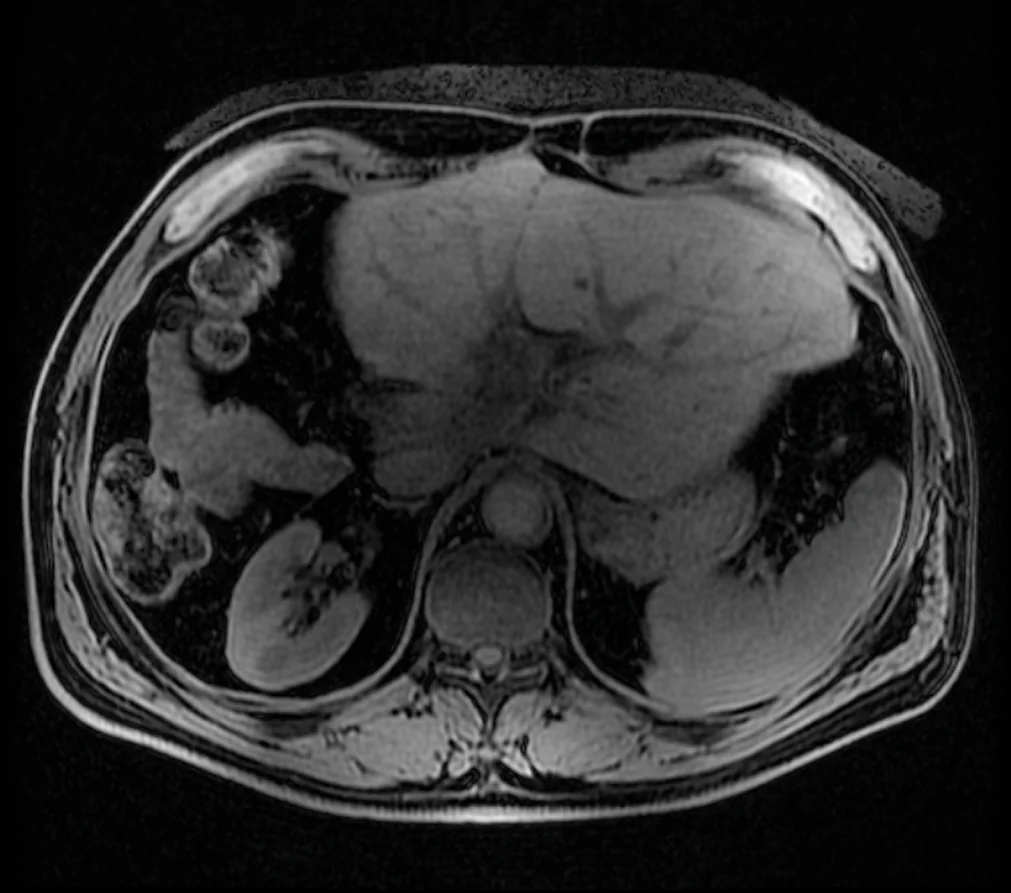
Postoperative surveillance magnetic resonance imaging/magnetic resonance cholangiopancreatography demonstrating postoperative anatomy after extended right hepatectomy, with no evidence of recurrent intraductal lesion and no biliary dilatation in the remnant left intrahepatic ducts.

## Discussion

When cross-sectional imaging is indeterminate^5^ and the hilar stricture cannot be crossed at ERCP, combining peroral SOC (for targeted histology at the obstruction) with antegrade pSOC (to inspect the upstream tree) can deliver definitive ductal mapping.^6^ In our case, antegrade pSOC demonstrated unilateral (right-sided) involvement with sparing of the left hepatic duct and its second-order branches, which was the key criterion to consider the lesion resectable and to proceed with ALPPS followed by extended right hepatectomy. The approach clarified true unilateral disease, reclassifying an “unresectable” perihilar lesion as resectable and guiding a tailored hepatectomy after ALPPS-induced hypertrophy. The workflow is practical in centers with integrated endoscopy and IR, may prevent both undertreatment and overtreatment, and can be performed within a single admission with tract maturation between sessions.^7^

Transhepatic percutaneous cholangioscopy/pSOC has a long track record for both diagnostic and therapeutic purposes.^8^ Diagnostic series report effective right/left mapping of intrahepatic ducts and targeted biopsies for indeterminate strictures and intraductal neoplasms when ERCP is limited.^9^^,^^10^ Therapeutically, pSOC has been used to clear difficult biliary stones, especially hepatolithiasis and complex intrahepatic calculi, via intraductal electrohydraulic lithotripsy or holmium:yttrium–aluminum–garnet laser lithotripsy, and has also been described for selective stricture dilation and stent retrieval in selected cases. These studies support our strategy of combining peroral SOC with antegrade pSOC to extend visualization beyond an uncrossable hilar stricture and to inform definitive management.

## Limitations

Limitations include the need for specialized equipment and coordinated expertise. Nonetheless, for borderline perihilar disease with equivocal imaging, complementary SOC routes provide a reliable path to determining resectability criteria for surgery. Although no adverse events occurred in our case, percutaneous SOC shares the adverse event profile of transhepatic biliary access, mainly cholangitis, hemobilia, bile leak, and drain-related issues and should, therefore, be performed in centers with IR and endoscopy expertise.

## Patient consent

This study was conducted in accordance with the Declaration of Helsinki. Written informed consent for procedures and publication was obtained.

## Disclosure

All authors disclosed no financial relationships.

## Supplementary data

Video 1 Single-operator cholangioscopy in the diagnosis and treatment planning of hilar intraductal papillary neoplasia of the bile duct: case report.

## References

[1] Sethi A., Tyberg A., Slivka A. (2022). Digital single-operator cholangioscopy improves interobserver agreement and accuracy for evaluation of indeterminate biliary strictures: the Monaco classification. J Clin Gastroenterol.

[2] Kahaleh M., Gaidhane M., Shahid H.M. (2022). Digital single-operator cholangioscopy interobserver study using a new classification: the Mendoza Classification (with video). Gastrointest Endosc.

[3] Milluzzo S.M., Landi R., Perri V. (2024). Diagnostic accuracy and interobserver agreement of cholangioscopy for indeterminate biliary strictures: a single-center experience. Dig Liver Dis.

[4] Sakai Y., Ohtsuka M., Sugiyama H. (2021). Current status of diagnosis and therapy for intraductal papillary neoplasm of the bile duct. World J Gastroenterol.

[5] Grigoriadis A. (2024). Improving our understanding of intraductal papillary neoplasms of the bile duct (IPNB). Eur Radiol.

[6] Lluís N., Serradilla-Martín M., Achalandabaso M. (2023). Intraductal papillary neoplasms of the bile duct: a European retrospective multicenter observational study (EUR-IPNB). Int J Surg.

[7] Wu R.-S., Liao W.-J., Ma J.-S. (2023). Epidemiology and outcome of individuals with intraductal papillary neoplasms of the bile duct. World J Gastrointest Oncol.

[8] Choi J.H., Lee S.K. (2013). Percutaneous transhepatic cholangioscopy: does its role still exist?. Clin Endosc.

[9] Du L., D'Souza P., Thiesen A. (2015). Percutaneous transhepatic cholangioscopy for indeterminate biliary strictures using the SpyGlass system: a case series. Endoscopy.

[10] Gerges C., Vázquez A.G., Tringali A. (2021). Percutaneous transhepatic cholangioscopy using a single-operator cholangioscope (pSOC), a retrospective, observational, multicenter study. Surg Endosc.

